# Fluoroscopic-Guided Barbotage and Corticosteroid Injection for the Treatment of Symptomatic Calcification of the Lateral Collateral Ligament: A Case Report

**DOI:** 10.7759/cureus.64407

**Published:** 2024-07-12

**Authors:** Emily Deschler, Enrique Galang

**Affiliations:** 1 Department of Anesthesiology/Comprehensive Pain and Spine Medicine, Sidney Kimmel Medical College at Thomas Jefferson University, Philadelphia, USA; 2 Department of Anesthesiology/Comprehensive Pain and Spine Medicine, Wake Forest University School of Medicine, Winston-Salem, USA

**Keywords:** calcification, barbotage, percutaneous needle aspiration and lavage, lateral knee pain, chronic knee pain, lateral collateral ligament

## Abstract

Periarticular calcification of the knee joint is a rare pathology that may be challenging to diagnose and manage when symptomatic. Here, we describe the first use of fluoroscopic-guided percutaneous needle aspiration and lavage (barbotage) with corticosteroid injection in a case of symptomatic calcification of the lateral collateral ligament (LCL). A 75-year-old female presented with acute lateral knee pain and stiffness, which subsequent radiographic imaging and diagnostic injection confirmed to be attributed to calcification within the LCL. Initial treatment with analgesic medications and a genicular nerve block failed to alleviate symptoms. However, complete resolution of symptoms was achieved following fluoroscopic-guided barbotage and steroid injection. This case underscores the importance of considering LCL calcification in the differential diagnosis of lateral knee pain. This case also illustrates the potential effectiveness of barbotage and adjunctive steroid injection as a minimally invasive treatment option for symptomatic LCL calcification, emphasizing the need for more rigorous studies evaluating treatment strategies for managing periarticular calcifications involving the knee joint.

## Introduction

Calcification of the lateral collateral ligament (LCL) is a rare cause of lateral knee pain. Calcification is characterized by the deposition of calcium hydroxyapatite crystals in periarticular soft tissues [1]. The precise etiology of calcification remains incompletely understood; however, various hypotheses propose that increased tension, degenerative changes, and tears or lesions may contribute as causal factors. Although most prevalent in the supraspinatus tendon of the shoulder, tendinous and ligamentous calcifications have been reported in other locations, including the hip, elbow, wrist, hand, neck, ankle, foot, and knee [2]. Among such cases, calcifications within soft tissue surrounding the knee joint are uncommon and there exist relatively few cases reported in the literature. Symptomatic calcifications of the knee have been described in the popliteus tendon, patellar tendon, LCL, medial collateral ligament, anterior cruciate ligament, and popliteofibular ligament-arcuate complex [3-8]. Given the rarity of symptomatic calcifications involving the knee joint and the scarcity of documented cases, there is a lack of consensus regarding effective treatment strategies.

It is estimated that up to 65% of known cases of periarticular calcification remain asymptomatic [1]. Conversely, symptomatic cases commonly present with pain and inflammation, potentially resulting in a restricted range of motion and functional impairment, which may mimic other pathologies and lead to misdiagnosis [1]. The majority of symptomatic calcifications exhibit favorable clinical responses to conservative treatment, such as analgesic anti-inflammatory medications and rest, typically resolving spontaneously. In refractory cases, interventions such as extracorporeal shock wave therapy (ESWT), barbotage, corticosteroid injection, or open surgical or arthroscopic excision of the calcification may offer effective therapeutic outcomes.

Barbotage is a safe and effective minimally invasive technique to remove calcific deposits [9]. The procedure utilizes image guidance to visualize the calcification, which is punctured with a needle and irrigated with saline to break down the calcification into fragments. The fragments are then aspirated for removal. Following barbotage, a small quantity of local anesthetic and corticosteroid may be injected. Several studies have demonstrated the safety and effectiveness of ultrasound-guided barbotage in the treatment of calcific tendonitis of the shoulder, with reductions in calcification size and subsequent clinical improvement [9,10]. Davis et al. reported the use of ultrasound-guided barbotage and steroid injection to successfully treat a case of lateral knee calcific tendonitis within the popliteofibular ligament-arcuate complex [8]. Here, we present a case describing the application of fluoroscopic-guided barbotage and steroid injection for the treatment of an acute calcification involving the LCL. To our knowledge, this is the first documented case of barbotage and steroid injection to successfully treat a symptomatic LCL calcification, with resultant functional improvement and resolution of pain.

## Case presentation

**Figure 1:**
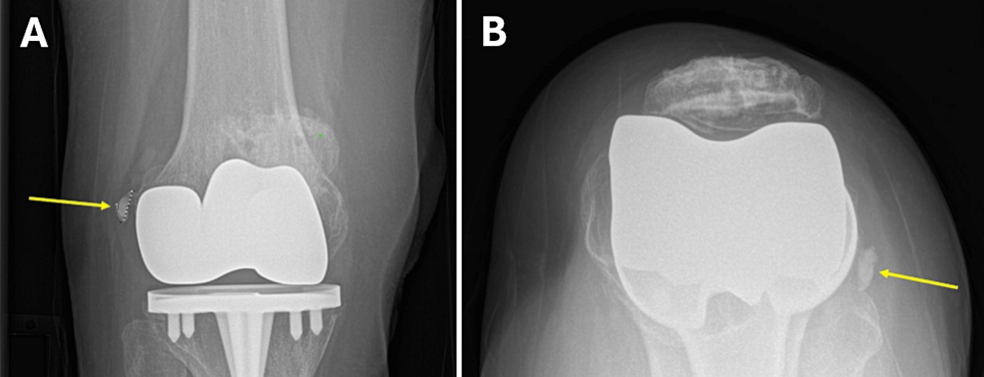
Radiographs showing a 14-mm curvilinear calcification adjacent to the lateral femoral condyle of the right knee (yellow arrow). (A) An anteroposterior radiographic view of the knee. (B) A Merchant radiographic view of the knee.

A 75-year-old female presented to our academic tertiary interventional pain medicine clinic with atraumatic, acute-onset lateral right knee pain with stiffness, with no known inciting event. The patient described the pain as achy and sore, localized over the lateral aspect of the knee. A physical examination of the right knee was negative for erythema, swelling, and signs of infection. The range of motion was within normal limits. No medial joint line tenderness or instability was noted. Relevant past medical history included a total right knee arthroplasty over 20 years prior. X-ray of the right knee revealed a 14-mm curvilinear calcification adjacent to the proximal lateral femoral condyle, possibly originating at the LCL (Figure 1). Supported by history, physical examination findings, radiographic imaging, a diagnostic injection of the right knee LCL that resulted in 75% pain relief for one week, and exclusion of other anatomic or medical pathology to explain symptoms, the patient was diagnosed with a symptomatic calcification of the LCL.

**Figure 2:**
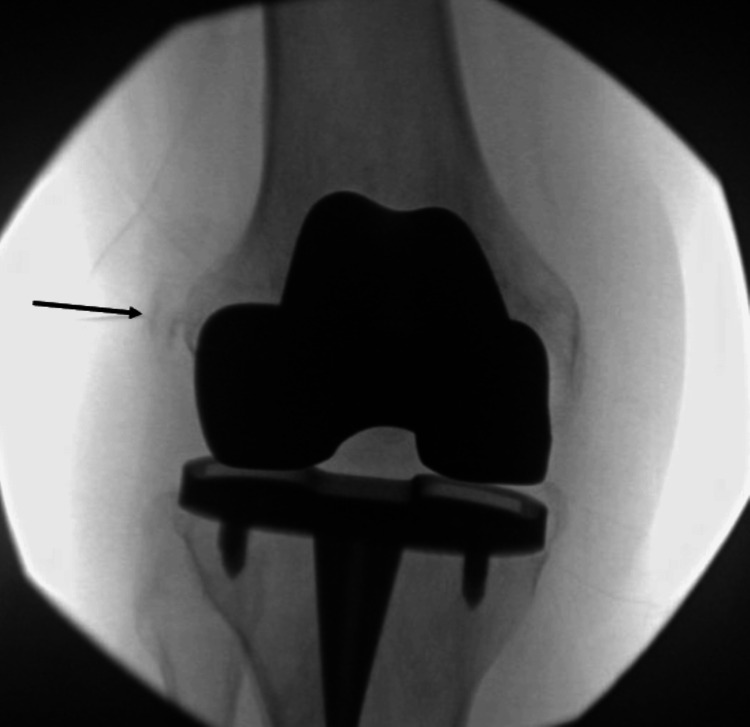
An anteroposterior fluoroscopic image identifying the calcification before the barbotage procedure (arrow).

One month following the initial treatment with a right knee genicular nerve radiofrequency ablation, the patient reported only 30% alleviation of the pain in the lateral aspect of the right knee coinciding with the calcification noted on prior X-rays. Six months after symptom onset, the patient experienced ongoing pain rated 7-8/10 on the Numeric Rating Scale, despite conservative treatment with analgesic medications. Therefore, the decision was made to proceed with fluoroscopic-guided barbotage and steroid injection of the LCL calcification. The calcification was first identified under live fluoroscopy (Figure 2). Superficial anesthesia was provided with 1% lidocaine. Next, a 1.5-inch, 25-gauge hypodermic needle was guided under live fluoroscopy to the calcific deposit, which was then aspirated. Following this, 3 mL of 0.25% bupivacaine and 5 mg of Depo-Medrol were incrementally injected, and the needle was then flushed with normal saline and withdrawn.

Following the procedure, the patient experienced significant pain relief. At the two-month follow-up, the patient reported 85% alleviation of pain with improved function. At the three-month follow-up, complete resolution of pain was achieved. Nine months post-barbotage, the patient continues to report the absence of pain, with no observed recurrence of the calcification.

## Discussion

Calcification of the LCL is an uncommon etiology of knee pain, with unknown pathogenesis of calcium deposition. Symptomatic LCL calcifications may present similarly to other knee joint pathologies such as gout, osteoarthritis, and iliotibial band syndrome. The diagnostic injection performed in our case confirmed that the calcification observed on radiographic imaging was the source of pain. Moreover, the lack of significant pain relief following the genicular nerve block indicated that osteoarthritis and other degenerative diseases were not the underlying cause of the pain. Most symptomatic calcifications typically respond well to conservative treatment and resolve spontaneously. However, in our case, symptoms persisted after six months of conservative management, leading to the consideration of more aggressive interventions.

Limited high-quality evidence exists regarding the efficacy of treatments for symptomatic calcifications, with most studies focusing exclusively on calcifications affecting the shoulder joint. At present, no high-level evidence studies involve patients with LCL calcification, as there exist only a few isolated case studies in the literature. A recent literature review identified 12 reports (16 cases) of LCL calcification documented in the English literature [5]. In nearly all cases, conservative treatment with anti-inflammatory and analgesic medications, rest, physical therapy, ESWT, and/or heat and cold therapy was the preferred first line of treatment. In nine cases successfully treated conservatively, pain relief was achieved within one to two weeks, with seven cases reporting resorption of the calcification within two weeks to five months. Four cases that failed conservative treatment opted for open surgical or arthroscopic excision of the calcification. One case utilized local steroid injection which failed to provide symptomatic improvement; nonetheless, the pain subsided two months later along with spontaneous resolution of the calcification. To our knowledge, our case is the first description of the successful use of barbotage together with steroid injection for the treatment of lateral knee pain associated with calcification in the LCL, with a subsequent decrease in pain and complete resolution of symptoms within three months post-barbotage.

In cases of tendon or ligament calcification that are non-responsive to conservative treatment, excision of the calcification generally serves as the subsequent treatment strategy, aiming to promptly and permanently remove the source of pain [11]. Various methods for calcification removal exist, including ESWT, barbotage, and open surgical or arthroscopic surgery. Nonoperative techniques are generally preferred over surgical or arthroscopic excision. Although surgical excision has demonstrated good clinical results, surgery involves a higher risk for infection and complications, longer hospital stays, longer rehabilitation and recovery periods, and greater healthcare costs. In contrast, several high-level evidence studies of patients with calcific tendonitis of the shoulder have shown that barbotage is a safe, simple, minimally invasive, cost-effective technique with high success rates and no major complications [9,10,12,13]. Consequently, these studies advocate for reserving surgical excision solely for cases in which barbotage fails to alleviate symptoms. Furthermore, ESWT is frequently employed as an alternative to surgical excision, as it is non-invasive, low-risk, and low-cost. However, randomized trials comparing barbotage and ESWT for the treatment of calcific tendonitis of the rotator cuff demonstrate that barbotage is more effective in pain relief, functional restoration, and elimination of calcific deposits compared to ESWT, and additional ESWT treatments are often required due to persistent symptoms [14,15].

Since the first application of barbotage under fluoroscopic guidance in 1978, numerous adaptations of the technique have emerged [16]. In 1995, Farin et al. introduced a modification to the technique by performing it under ultrasound guidance and demonstrated comparable clinical effectiveness in treating rotator cuff calcific tendonitis while mitigating radiation exposure of fluoroscopic guidance [11,17]. Although ultrasonography is increasingly employed for barbotage procedures, in our case, we opted for barbotage guided by fluoroscopy. Fluoroscopy was utilized as the calcification was readily identifiable and there was a potential concern for artifacts utilizing ultrasound given the patient’s history of knee replacement.

Another variation in the barbotage technique and a topic of debate is the administration of corticosteroid injection as an adjunct treatment after barbotage. In our case, the application of corticosteroid injection following barbotage resulted in favorable clinical outcomes, with no recurrence of the calcification observed. Comfort et al. advised against the use of local corticosteroid injection either as a primary procedure or as an adjunct after barbotage due to the belief that corticosteroids might abort the natural course of the disease and promote recurrences [16]. Furthermore, Farin et al. asserted that post-barbotage injection of water-soluble cortisone into the subacromial-subdeltoid bursa might prevent adhesive capsulitis of the shoulder [17]. However, they acknowledged the potential for cortisone to impede the resorption of calcific deposits and induce tendon weakening, thereby increasing susceptibility to tearing. Nevertheless, these drawbacks were deemed minimal when cortisone was administered in a water-soluble form and targeted into the bursal system. In addition, a review article on fluoroscopic-guided barbotage for calcific tendonitis of the shoulder claimed that a key objective of the procedure is to reduce inflammation secondary to residual calcific deposits by in situ corticosteroid injection [13].

Several recent studies have attempted to evaluate the therapeutic value of post-barbotage steroid injection, yielding conflicting outcomes. A randomized non-inferiority trial by Darriertort-Laffite et al. aimed to determine whether a saline solution is non-inferior to steroids in preventing acute pain reactions following barbotage for shoulder calcific tendonitis [18]. Despite inconclusive findings, the trial highlighted that post-barbotage steroid injection significantly improves pain and function with no significant effect on calcium resorption. Conversely, Malahias et al. illustrated in a randomized trial that the supplementary administration of corticosteroid injection following barbotage does not confer any additional short- to mid-term therapeutic benefit when compared to barbotage alone in patients with shoulder calcific tendinosis [19]. However, de Witte et al. found in a randomized controlled trial that both barbotage with steroid injection and isolated steroid injection lead to clinical improvement at one-year follow-up, with superior clinical and radiographic results observed in the barbotage group [12]. Intriguingly, a recent randomized sham-controlled trial by Moosmayer et al. revealed that treatment benefits derived from barbotage with steroid injection or from steroid injection alone are not superior to those from sham treatment in patients with rotator cuff calcific tendinopathy, challenging the efficacy of these interventions for calcific tendonitis [20]. Consequently, the therapeutic value of corticosteroid injection post-barbotage remains controversial.

Of note, clinical studies assessing the efficacy of barbotage are predominantly confined to calcifications affecting the shoulder, given its higher incidence in this location. Caution is warranted when extrapolating the findings of these studies to cases involving the knee joint. Rigorous randomized controlled trials directly comparing the efficacy of barbotage with and without steroid injection for managing LCL calcification, along with studies comparing barbotage to alternative interventions such as steroid injection alone and surgical excision, would provide valuable insights. However, the relatively low prevalence of LCL calcification may pose challenges in conducting such studies. Nonetheless, based on our experience with this case, we suggest considering barbotage and steroid injection as a viable therapeutic approach in managing symptomatic LCL calcifications, potentially offering an alternative to surgical intervention and its associated risks.

## Conclusions

Symptomatic calcification of the LCL of the knee represents a rare cause of lateral knee pain and may be challenging to diagnose due to its resemblance to other knee joint pathologies. LCL calcification should be considered a differential diagnosis for patients experiencing lateral knee pain. While conservative treatments are often effective, refractory cases may necessitate more aggressive interventions. Barbotage has shown promising results in managing symptomatic calcifications, particularly in the shoulder joint. However, its application in LCL calcification cases remains unexplored, highlighting the need for further research to establish its efficacy in the knee joint. Additionally, the adjunctive use of corticosteroid injections post-barbotage lacks consensus, necessitating more comprehensive investigations. Despite the challenges posed by the rarity of LCL calcification cases, our case underscores the potential of barbotage and steroid injection as an effective therapeutic option, offering a middle ground between conservative and surgical approaches. Future studies, including rigorous randomized controlled trials directly comparing different treatment modalities, are warranted to enhance our understanding and optimize management strategies for symptomatic LCL calcifications.
